# Supplementary material for: Comparative Efficacy of Tyrosine Kinase Inhibitors and Antibody–Drug Conjugates in HER2-Positive Metastatic Breast Cancer Patients with Brain Metastases: A Systematic Review and Network Meta-Analysis
Source: Cancers (Basel). 2022 Jul 11;14(14):3372. doi: 10.3390/cancers14143372 (PMC9321046; doi:10.3390/cancers14143372)
Supplement: Supplementary file 1 [file cancers-14-03372-s001.zip › cancers-1805528-supplementary.pdf]

cancers

MDPI

Review

Comparative Efficacy of Tyrosine Kinase Inhibitors and Antibody–Drug Conjugates in HER2-Positive Metastatic Breast Cancer Patients with Brain Metastases: A Systematic Review and Network Meta-Analysis

Yan Wang, Hangcheng Xu, Yiqun Han, Yun Wu and Jiayu Wang

Supplementary Materials

Table S1. Detailed searching strategies.

a	Search strategy in PubMed, May 5th, 2022. (n=1471)	**Search terms:** (breast OR mammary) AND (cancer OR carcinoma OR malignancy OR neoplasm OR tumor) AND (HER-2 OR HER2 OR neu OR ERBB2 OR human epidermal growth factor receptor 2) AND (positive OR enriched OR overexpressing OR overexpressed) AND (metastasis OR metastatic OR advanced OR secondary OR recurrent OR inoperable OR unresectable OR disseminated OR incurable OR Stage 3 OR Stage 4 OR Stage III OR Stage IV) AND (lapatinib OR neratinib OR pyrotinib OR tucatinib OR poziotinib OR afatinib OR sunitinib OR dasatinib OR trastuzumab emtansine OR T-DM1 OR trastuzumab-DM1 OR trastuzumab-MCC-DM1 OR trastuzumab deruxtecan OR DS8201 OR trastuzumab duocarmazine OR SYD985)
b	Search strategy in PubMed, May 5th, 2022. (n=1471)	**Search terms:** (breast OR mammary) AND (cancer OR carcinoma OR malignancy OR neoplasm OR tumor) AND (HER-2 OR HER2 OR neu OR ERBB2 OR human epidermal growth factor receptor 2) AND (positive OR enriched OR overexpressing OR overexpressed) AND (metastasis OR metastatic OR advanced OR secondary OR recurrent OR inoperable OR unresectable OR disseminated OR incurable OR Stage 3 OR Stage 4 OR Stage III OR Stage IV) AND (lapatinib OR neratinib OR pyrotinib OR tucatinib OR poziotinib OR afatinib OR sunitinib OR dasatinib OR trastuzumab emtansine OR T-DM1 OR trastuzumab-DM1 OR trastuzumab-MCC-DM1 OR trastuzumab deruxtecan OR DS8201 OR trastuzumab duocarmazine OR SYD985)
c	Search strategy in Embase, May 5th, 2022. (n=2463)	**Search terms:** ('breast':ab,ti OR 'mammary':ab,ti) AND ('cancer':ab,ti OR 'carcinoma':ab,ti OR 'malignancy':ab,ti OR 'neoplasm':ab,ti OR 'tumor':ab,ti) AND ('her-2':ab,ti OR 'her2':ab,ti OR 'neu':ab,ti OR 'erbb2':ab,ti OR 'human epidermal growth factor receptor 2':ab,ti) AND ('positive':ab,ti OR 'enriched':ab,ti OR 'overexpressing':ab,ti OR 'overexpressed':ab,ti) AND ('metastasis':ab,ti OR 'metastatic':ab,ti OR 'advanced':ab,ti OR 'secondary':ab,ti OR 'recurrent':ab,ti OR 'inoperable':ab,ti OR 'unresectable':ab,ti OR 'disseminated':ab,ti OR 'incurable':ab,ti OR 'stage 3':ab,ti OR 'stage 4':ab,ti OR 'stage iii':ab,ti OR 'stage iv':ab,ti) AND ('lapatinib':ab,ti OR 'neratinib':ab,ti OR 'pyrotinib':ab,ti OR 'tucatinib':ab,ti OR 'poziotinib':ab,ti OR 'afatinib':ab,ti OR 'sunitinib':ab,ti OR 'dasatinib':ab,ti OR 'trastuzumab emtansine':ab,ti OR 't-dm1':ab,ti OR 'trastuzumab-dm1':ab,ti OR 'trastuzumab-mcc-dm1':ab,ti OR 'trastuzumab deruxtecan':ab,ti OR 'ds8201':ab,ti OR 'trastuzumab duocarmazine':ab,ti OR 'syd985':ab,ti)
d	Search strategy in the Cochrane Library, May 5th, 2022. (n=729)	**Search terms:**<br>#1 MeSH descriptor: [Breast Neoplasms] explode all trees<br>#2 (breast):ti,ab,kw (Word variations have been searched)<br>#3 (mammary):ti,ab,kw (Word variations have been searched)<br>#4 #2 OR #3<br>#5 (cancer):ti,ab,kw (Word variations have been searched)<br>#6 (carcinoma):ti,ab,kw (Word variations have been searched)<br>#7 (malignancy):ti,ab,kw (Word variations have been searched)<br>#8 (neoplasm):ti,ab,kw (Word variations have been searched)<br>#9 (tumor):ti,ab,kw (Word variations have been searched)<br>#10 #5 OR #6 OR #7 OR #8 OR #9<br>#11 #4 AND #10<br>#12 #1 OR #11<br>#13 MeSH descriptor: [Receptor, ErbB-2] explode all trees<br>#14 (HER-2):ti,ab,kw (Word variations have been searched)<br>#15 (HER2):ti,ab,kw (Word variations have been searched)<br>#16 (neu):ti,ab,kw (Word variations have been searched)

#17 (ERBB2):ti,ab,kw (Word variations have been searched)
#18 (human epidermal growth factor receptor 2):ti,ab,kw (Word variations have been searched)
#19 #13 OR #14 OR #15 OR #16 OR #17 OR #18
#20 (positive):ti,ab,kw (Word variations have been searched)
#21 (enriched):ti,ab,kw (Word variations have been searched)
#22 (overexpressing):ti,ab,kw (Word variations have been searched)
#23 (overexpressed):ti,ab,kw (Word variations have been searched)
#24 #20 OR #21 OR #22 OR #23
#25 MeSH descriptor: [Neoplasm Metastasis] explode all trees
#26 (metastasis):ti,ab,kw (Word variations have been searched)
#27 (metastatic):ti,ab,kw (Word variations have been searched)
#28 (advanced):ti,ab,kw (Word variations have been searched)
#29 (secondary):ti,ab,kw (Word variations have been searched)
#30 (recurrent):ti,ab,kw (Word variations have been searched)
#31 (inoperable):ti,ab,kw (Word variations have been searched)
#32 (unresectable):ti,ab,kw (Word variations have been searched)
#33 (disseminated):ti,ab,kw (Word variations have been searched)
#34 (incurable):ti,ab,kw (Word variations have been searched)
#35 (Stage 3):ti,ab,kw (Word variations have been searched)
#36 (Stage 4):ti,ab,kw (Word variations have been searched)
#37 (Stage III):ti,ab,kw (Word variations have been searched)
#38 (Stage IV):ti,ab,kw (Word variations have been searched)
#39 #25 OR #26 OR #27 OR #28 OR #29 OR #30 OR #31 OR #32 OR #33 OR #34 OR #35 OR #36 OR #37 OR #38
#40 MeSH descriptor: [Lapatinib] explode all trees
#41 (lapatinib):ti,ab,kw (Word variations have been searched)
#42 (neratinib):ti,ab,kw (Word variations have been searched)
#43 (pyrotinib):ti,ab,kw (Word variations have been searched)
#44 (tucatinib):ti,ab,kw (Word variations have been searched)
#45 (poziotinib):ti,ab,kw (Word variations have been searched)
#46 (afatinib):ti,ab,kw (Word variations have been searched)
#47 MeSH descriptor: [Afatinib] explode all trees
#48 MeSH descriptor: [Sunitinib] explode all trees
#49 (sunitinib):ti,ab,kw (Word variations have been searched)
#50 MeSH descriptor: [Dasatinib] explode all trees
#51 (dasatinib):ti,ab,kw (Word variations have been searched)
#52 MeSH descriptor: [Ado-Trastuzumab Emtansine] explode all trees
#53 (trastuzumab emtansine):ti,ab,kw (Word variations have been searched)
#54 (T-DM1):ti,ab,kw (Word variations have been searched)
#55 (trastuzumab-DM1):ti,ab,kw (Word variations have been searched)
#56 (trastuzumab-MCC-DM1):ti,ab,kw (Word variations have been searched)
#57 (trastuzumab deruxtecan):ti,ab,kw (Word variations have been searched)
#58 (DS8201):ti,ab,kw (Word variations have been searched)
#59 (trastuzumab duocarmazine):ti,ab,kw (Word variations have been searched)
#60 (SYD985):ti,ab,kw (Word variations have been searched)
#61 #40 OR #41 OR #42 OR #43 OR #44 OR #45 OR #46 OR #47 OR #48 OR #49 OR #50 OR #51 OR #52 OR #53 OR #54 OR #55 OR #56 OR #57 OR #58 OR #59 OR #60
#62 #12 AND #19 AND #24 AND #39 AND #61

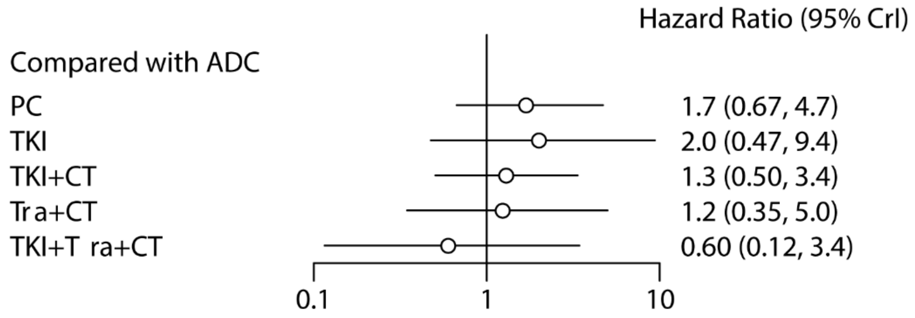

Figure S1. PFS comparisons between TKI-containing regimens and ADC. (I^2=15%).

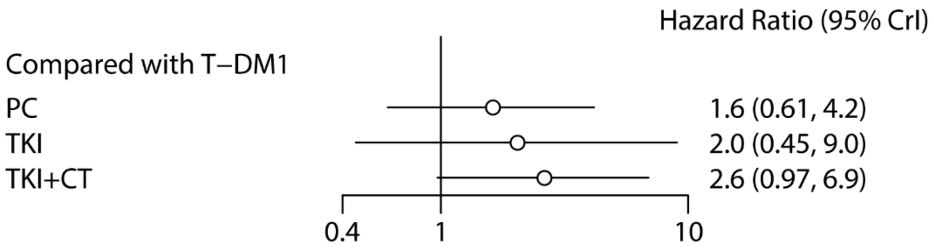

Figure S2. OS comparisons between TKI-containing regimens and T-DM1. (I^2=13%).

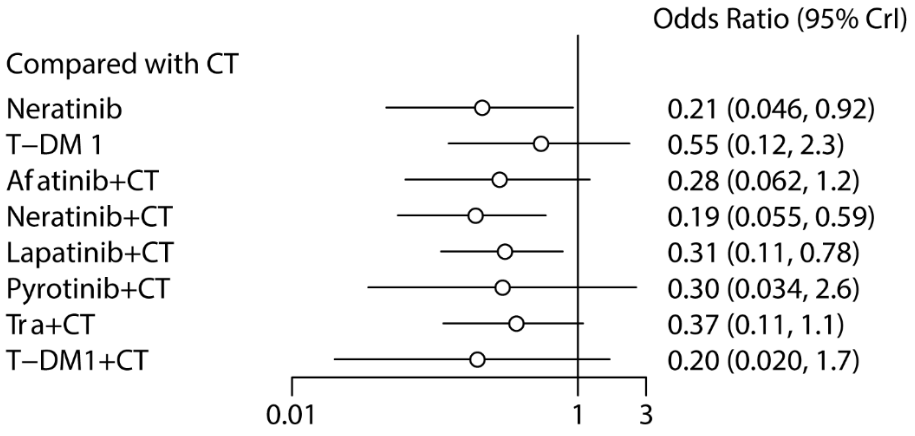

Figure S3. CNS progression rate comparisons among different therapeutic strategies regardless of the baseline BCBM status. (I^2=0%).

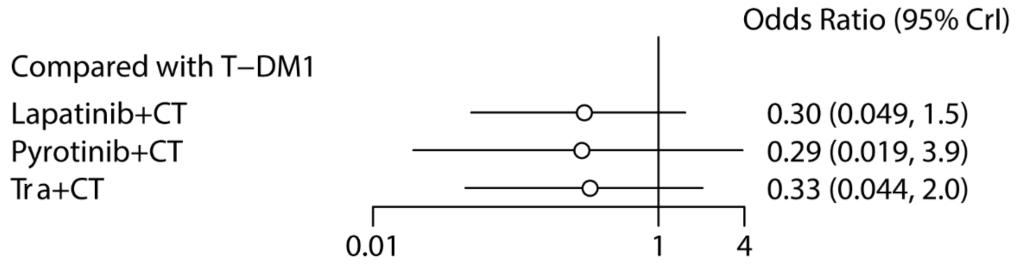

Figure S4. CNS progression rate comparisons in patients without baseline BCBM. (I^2=12%).

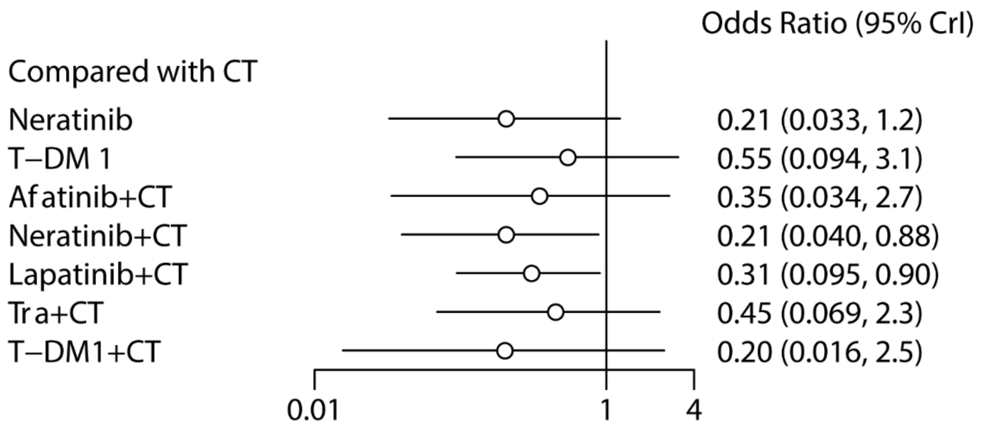

Figure S5. CNS progression rate comparisons in patients with non or stable BCBM in baseline. (I^2=0%).
